# Cell cycle regulation by long non-coding RNAs

**DOI:** 10.1007/s00018-013-1423-0

**Published:** 2013-07-24

**Authors:** Masatoshi Kitagawa, Kyoko Kitagawa, Yojiro Kotake, Hiroyuki Niida, Tatsuya Ohhata

**Affiliations:** 1 Department of Molecular Biology, Hamamatsu University School of Medicine, 1-20-1 Handayama, Higashi-ku, Hamamatsu, 431-3125 Japan; 2 Department of Biological and Environmental Chemistry, Faculty of Humanity-Oriented Science and Engineering, Kinki University, 11-6 Kayanomori, Iizuka, Fukuoka, 820-8555 Japan

**Keywords:** lncRNA, DNA damage response, Cyclin-CDK, CDK inhibitor, pRB, p53

## Abstract

The mammalian cell cycle is precisely controlled by cyclin-dependent kinases (CDKs) and related pathways such as the RB and p53 pathways. Recent research on long non-coding RNAs (lncRNAs) indicates that many lncRNAs are involved in the regulation of critical cell cycle regulators such as the cyclins, CDKs, CDK inhibitors, pRB, and p53. These lncRNAs act as epigenetic regulators, transcription factor regulators, post-transcription regulators, and protein scaffolds. These cell cycle-regulated lncRNAs mainly control cellular levels of cell cycle regulators via various mechanisms, and may provide diversity and reliability to the general cell cycle. Interestingly, several lncRNAs are induced by DNA damage and participate in cell cycle arrest or induction of apoptosis as DNA damage responses. Therefore, deregulations of these cell cycle regulatory lncRNAs may be involved in tumorigenesis, and they are novel candidate molecular targets for cancer therapy and diagnosis.

## Introduction

**Fig. 1:**
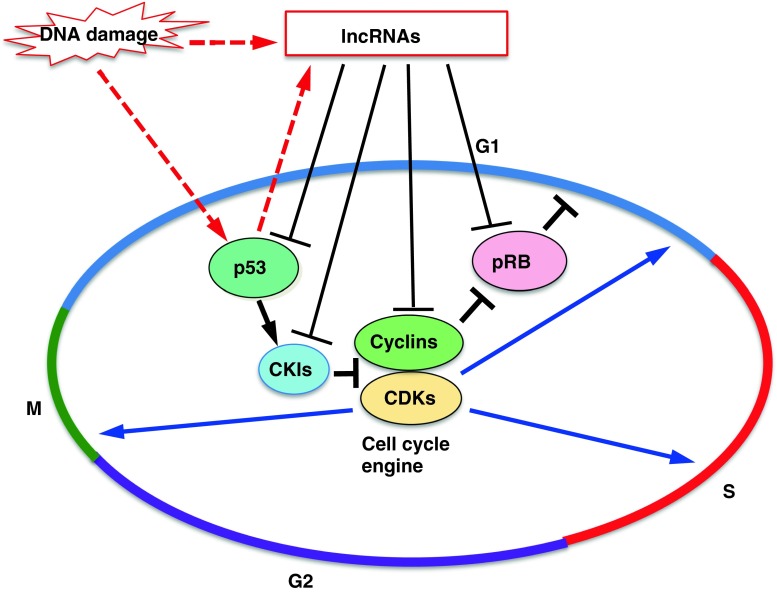
Outline of cell cycle control and involvement of lncRNAs. The mammalian cell cycle is controlled by cyclin-dependent kinases (CDKs) and their related pathways. CDKs are activated via binding to their selected cyclins in specific phases of the cell cycle, following which they phosphorylate their target proteins. The CDK inhibitors (CKIs) negatively regulate the activities of CDKs and control the cell cycle. pRB regulates G1/S progression. The p53 pathway plays a role in DNA damage response as a gatekeeper of the genome. Several lncRNAs control the expression of cyclins-CDKs, CKIs, pRB and p53, and participate in cell cycle regulation. Some of these lncRNAs are induced by DNA damage and inhibit cell cycle progression by regulating these cell cycle regulators

The mammalian cell cycle is controlled by cyclin-dependent kinases (CDKs) and their related pathways (Fig. 1) [1, 2]. The CDKs, particularly CDK1, CDK2, and CDK4/6, are activated via binding to their selected cyclins, including cyclins A, B, D, and E, in specific phases of the cell cycle, following which they phosphorylate their target proteins to enable cell cycle progression. The activities of the CDKs are controlled not only by cyclins but also by phosphorylation or dephosphorylation by Wee1 kinase or CDC25 phosphatase [1]. Moreover, CDK inhibitors including p15^*ink4b*^, p16 ^*ink4a*^, p18 ^*ink4d*^, p21^*Cip1*^, p27 ^*Kip1*^, and p57 ^*Kip2*^ specifically bind to their target cyclin–CDK complexes and inhibit their activities to negatively regulate the cell cycle [3–5].

CDKs and their related pathways control the cell cycle by maintaining exit and entry to the different phases of the cell cycle. In the G1 phase, growth stimuli such as growth factors often activate the MAP kinase pathway, following which genes encoding the cyclin Ds are transcribed. The resulting products bind to and activate CDK4/6 [6]. Cyclin Ds–CDK4/6 complexes phosphorylate retinoblastoma protein (pRB) and its family members, p107 and p130, in the late G1 phase and activate E2F-mediated transcription, which induces the expression of several growth-promoting genes [7, 8]. At the G1/S transition point, cyclin E-CDK2 phosphorylates pRB as well as several proteins involved in DNA replication to promote G1/S progression [9]. Cyclin B-CDK1 has many targets including APC/cyclosome, and promotes maturation of the G2 phase and critically participates in M phase events [10].

The cellular levels of cell cycle regulators such as cyclins, CDKs, CDK inhibitors, CDC25, RB, and E2F are critical for cell cycle regulation. After the cell cycle regulators complete their functions, they are ubiquitylated by specific E3 ligases and eliminated via the ubiquitin–proteasome pathway [11–13]. The level of cell cycle regulators is precisely controlled by not only post-translational but also translational mechanisms. For example, several micro-RNAs (miRNAs) participate in cell cycle regulation through translational regulation [14]. MiRNAs are small non-coding RNA molecules containing 22 nucleotides, and negatively regulate translation through binding of the untranslated region of its target mRNAs [15]. The let-7 miRNA family negatively regulates cyclins A and D, and CDK4/6 and CDC25A [16]. The miR-15 family also inhibits the translation of cyclin D, CDK4, and CDC27 [17, 18]. Interestingly, these let-7 and miR-15 family members may be involved in tumorigenesis since they are downregulated in various human cancers [16–18]. Alternatively, cyclin D1 is a target for not only let-7 and miR-15 miRNAs but also miR-19a, 26a, and 34a [15]. Furthermore, p27 ^*Kip1*^ is targeted for regulation by the miR-181 family [19] and the miR-221 family [20]. The roles of other miRNAs in the expression of cell cycle regulators have also been reported [15]. Thus, it has been shown that the cell cycle regulators are critically and precisely controlled by E3 ligases and miRNAs both post-translationally and at the translational level.

Here, we focus on long non-coding RNAs (lncRNAs) involved in the regulation of the cell cycle through their various functions as epigenetic regulators, transcription factor regulators, post-transcription regulators and protein scaffolds [21, 22]. LncRNAs are non-protein coding transcripts longer than 200 nucleotides, and can be divided into at least five categories based on their structural characteristics, including intergenic lncRNAs (lincRNAs), intronic lncRNAs, natural antisense transcripts, pseudogenes, and retrotransposons [23]. Recent mass-scale transcriptome analysis has revealed that many kinds of lncRNAs are transcribed in large amounts in the eukaryotic genome [24]. However, the question remains as to whether these lncRNAs are merely by-products of the transcriptional units or have a critical function for biological processes. However, it has become clear that some of these lncRNAs participate in various biological processes such as genome imprinting, X-inactivation, development, differentiation, and cell cycle regulation [22, 24–26]. For example, *HOTAIR*, a well-investigated lncRNA, is involved in correct development and tumorigenesis through recruiting the polycomb group (PcG) complex to its targeted *HOX* genes for their repression [26, 27]. The PcG complex contributes to the epigenetic regulation of its target genes by forming Polycomb repressive complex 1 (PRC1) and 2 (PRC2). PRC2 participates in histone H3K27 methylation and, following histone H2AK119 monoubiquitination by PRC1, collaboratively represses target gene transcription. In addition to *HOTAIR*, several lncRNAs such as *XIST*, *AIR*, and *KCNQ1OT1* also recruit chromatin modifiers including PcG and H3K9 methyltransferase G9a to their target loci [25, 28–30]. Moreover, *ANRIL* (antisense non-coding RNA in the *INK4* locus) directly binds to PcGs and recruits them to the *INK4* locus to promote gene silencing [31, 32]. Thus, *HOTAIR, XIST, AIR, KCNQ1OT1*, and *ANRIL* function as epigenetic regulators by negatively regulating target gene transcription through recruitment of chromatin modifiers. Recently, several lncRNAs that participate in the expression of several cell cycle regulators have been reported (summarized in Fig. 1; Table 1). In this review, we introduce these lncRNAs and discuss their functions in cell cycle regulation.

**Table 1:** LncRNAs involved in the cell cycle control

lncRNA	How it is induced	The effects of the lncRNA on its targets in cell cycle (phase)	References
*ncRNA*_*CCNDI*_	DNA damage	Suppression of *Cyclin D1* transcription with TLS (G1)	[33, 34]
*gadd7*	DNA damage	Destabilization of *CDK6 mRNA* (G1)	[35, 36]
*MALAT1*	High expression In cancer	Promotion of cell-cycle regulators such as cyclin A2 and B1 (G1 and G2/M)	[37–39]
*SRA*	?	Suppression of *Cyclin A, B cdc20. Cdt1* transcription (G1 and G2/M)	[40, 41]
*ANRIL*	DNA damage	Suppression of *p15/p16* transcription with PRC1/2 (G1)	[31, 32, 52]
*lncRNA*-*HEIH*	High expression In HCC	Suppression of *p16. p21, p27 and p57* transcription with PRC2(G0/G1)	[53]
*HULC*	HBx-mediated	Suppression of *p18* expression (G1)	[60–62]
*KCNQ10T1*	Paternal expression	Suppression of *p57* transcription with PRC2 and G9a (G1?}	[64]
*H19 lncRNA*	E2F1-mediated	Downregulation of *RB mRNA* via *miR675* (G1)	[69–71]
*lncRNA*-*RoR*	p53-mediated	Suppression of *p53 mRNA* translation (G2/M)	[74]
*p53*-*induced eRNA*	p53-mediated	Promotion of p53 target genes transcription (G1?)	[75]
*loc285194*	p53-mediated	Growth inhibition by suppression of miR211 (G1?)	[76]
*lncRNA*-*p21*	p53-mediated	Suppression of transcription of the target genes involved in apoptosis and cell cycle with hnRNA-K (G1?) suppression of β-catenin and *Jun B mRNA* translation	[77, 78]
*PANDA*	DNA damage	Suppression of *FAS* and *BIK* transcription (G1?)	[79]

## LncRNAs regulating cyclins and CDKs

**Fig. 2:**
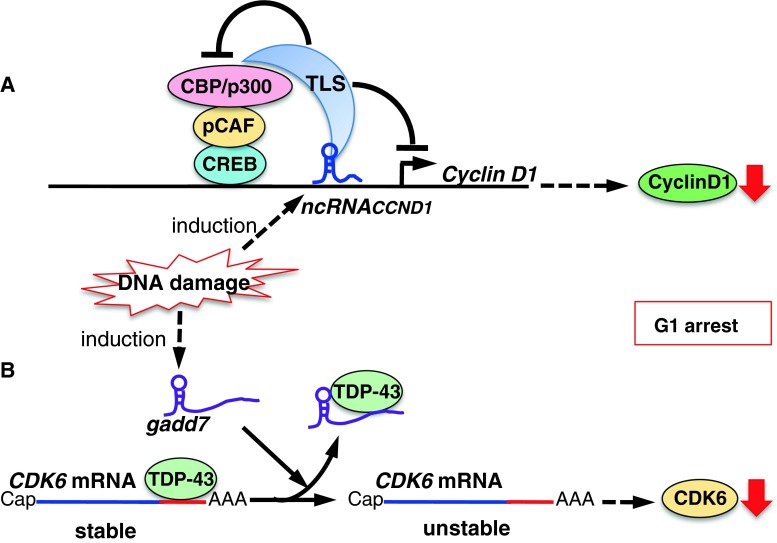
Model showing the proposed mechanisms of lncRNA-mediated regulation of cyclin D1 (**a**) and CDK6 (**b**) induced by DNA damage. **a** DNA damage induces the transcription of *ncRNA*_*CCND1*_ from the promoter region of the *cyclin D1* gene. *ncRNA*_*CCND1*_ associates with and recruits TLS, an RNA binding protein, to the *cyclin D1* promoter. The *ncRNA*_*CCND1*_–TLS complex inhibits the CBP/p300–pCAF–CREB coactivator complex and thereby prevents *cyclin D1* gene transcription. **b** DNA damage induces the expression of the lncRNA, *gadd7*, which dissociates TDP-43 from the CDK6 mRNA to destabilize it, and CDK6 is thereby downregulated, inhibiting the G1/S transition. The lncRNAs *gadd7* and *ncRNA*_*CCND1*_ may collaboratively participate in the G1 checkpoint in response to DNA damage

Cyclins and CDKs are key players in cell cycle regulation (Fig. 1). *NcRNA*_*CCND1*_, also called pncRNA (promoter-associated non-coding RNA), is transcribed from the upstream region of the *cyclin D1* gene, *CCND1*, and negatively regulates cyclin D1. *NcRNA*_*CCND1*_ functions as a transcription factor regulator [33]. It is induced in a DNA damage-dependent manner, and associates with and recruits TLS (translocated in liposarcoma) [34], an RNA binding protein. The *ncRNA*_*CCND1*_-TLS complex is recruited to the *CCND1* promoter to inhibit the activity of the coactivator, CBP/p300, thereby preventing *CCND1* transcription (Fig. 2a). Thus, suppression of cyclin D1 regulated by the *ncRNA*_*CCND1*_-TLS complex may participate in G1 arrest in response to DNA damage.

*Gadd7* is an lncRNA involved in regulating CDK6 expression [35] in a posttranscriptional manner. TDP-43 (TAR DNA binding protein) binds to the 3′ untranslated region of *CDK6* mRNA to stabilize it. *Gadd7* is transcriptionally induced via DNA damage mediated by UV and cisplatin [35, 36], and binds to TDP-43 and dissociates from *CDK6* mRNA. The *CDK6* mRNA is then degraded, resulting in inhibition of the G1/S transition (Fig. 2b). Therefore, *gadd7* negatively controls CDK6 expression, functioning as a translation regulator. Interestingly, *gadd7* specifically controls mRNA stability for *CDK6*, but not *CDK4*, *CDK2*, or *CCND1*, by trapping TDP-43. The physiological relevance of the selective suppression of *CDK6* remains to be determined. *Gadd7* may be involved in the G1 checkpoint by collaborating with the lncRNA, *ncRNA*_*CCND1*_, to downregulate the cyclin D1–CDK6 complex, thereby arresting cell cycle progression in response to DNA damage (Fig. 2a, b). This may represent a novel G1-checkpoint cascade, but further studies are required.

*MALAT1*, an mRNA splicing mediator [37], is upregulated in several human cancers and contributes to cancer cell proliferation [38]. *MALAT1* depletion results in arrest at G1 and promotes expression of p53 as well as p16, p21, and p27 in human fibroblasts [39] (Table 1). In contrast, *MALAT1* depletion suppresses various genes involved in cell cycle progression such as the genes encoding cyclin A2 and Cdc25A, thereby arresting the cell cycle in G1. Moreover, in G2/M progression, *MALAT1* is required for expression of B-Myb, which is involved in the expression of mitotic proteins such as cyclin B1, CDK1, FoxoM1, and PLK by controlling the splicing of B-Myb mRNA [39]. Therefore, *MALAT1* may contribute to cell cycle progression in each phase by coordinated control of cell cycle regulators.

Steroid receptor RNA activator (*SRA*) was identified as an lncRNA that binds to steroid receptors [40]. *SRA* forms the SRC-1 complex to activate transcription, mediated by steroid receptors such as progesterone receptor and estrogen receptor. It also binds to various other proteins such as myoD, and has multiple cellular functions such as myogenesis. *SRA* also binds to PPARγ and coactivates gene expression mediated by PPARγ. As such, *SRA* regulates adipogenesis and insulin sensitivity via PPARγ [41]. Additionally, *SRA* shows PPARγ-independent activity. Overexpression of *SRA* in pre-adipocytes downregulates the expression of cell cycle-promoting genes such as those encoding the cyclins [cyclins (A2, B1/2)], *CDC20*, *MCMs* (*3, 4, 5, 6*), and *CDT1*. Conversely, these genes are upregulated by depletion of *SRA*. However, it remains to be elucidated whether *SRA* directly or indirectly suppresses the transcription of these genes, and further investigation into the mechanisms of *SRA*-regulated gene expression is required.

## LncRNAs regulating CDK inhibitors

### INK4 family inhibitors

The CDK inhibitory proteins, p16^ink4a^ and p15^ink4b^ (hereafter p16 and p15), bind to and inhibit CDK4 and 6, respectively, via their ankyrin repeats [3, 42]. The p15 and p16 genes (*CDKN2B* and *CDKN2A*, respectively) are located at the *INK4* locus together with the alternating reading frame gene, *ARF* [42]. ARF inhibits MDM2-dependent degradation of both p53 [43] and pRB [44]. Therefore, the expression of *INK4* locus genes is critical for cell cycle regulation. The INK4 proteins are relatively stable, and their ubiquitin-dependent proteolysis is not particularly important for controlling their cellular levels. Therefore, the *INK4* locus genes are mainly regulated by transcription. The participation of several transcription factors, including the ETS family [45], FOXO [46], and SP1 [47], has been reported. Moreover, the locus is regulated epigenetically. It has been suggested that PU.1 cooperates with DNA methyltransferase and is involved in the *INK4* locus via methylation of CpG islands [48]. Moreover, PcG is recruited to the *INK4* locus, thereby suppressing transcription via histone H3K27 methylation [49].

**Fig. 3:**
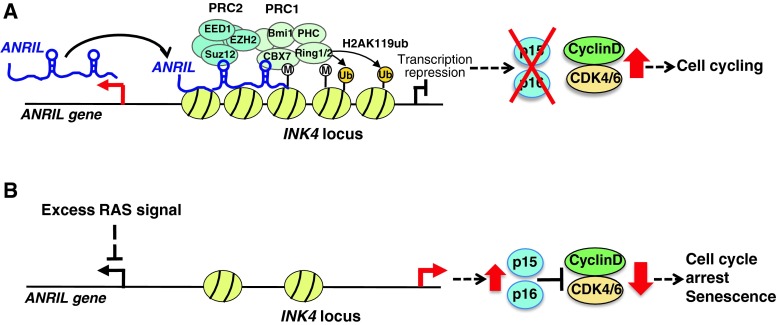
Model showing the proposed mechanisms of *ANRIL*-mediated regulation of the *INK4* locus. **a** Model of *ANRIL*-mediated repression of the *INK4* locus. *ANRIL* binds to the PRC2 complex to recruit it to the *INK4* locus. Then, histone H3K27 methylation (M) is mediated by EZH2 in the PRC2 complex with *ANRIL*, which is recognized by CBX7 to recruit the PRC1 complex to the region. Histone H2AK119 monoubiquitination (Ub) is thereby induced to repress the transcription of *INK4*. **b** Excess RAS signaling suppresses the expression of *ANRIL*. Overexpression of activated H-RasG12V in WI38 fibroblasts promotes excess RAS signaling and suppresses *ANRIL* expression. Then, p15 and p16 are induced and the cell cycle undergoes arrest, inducing a premature senescence-like phenotype

It has been suggested that antisense RNA transcribed near the *p15* gene controls transcription of *p15* [50]. Pasmant et al. identified an lncRNA, *ANRIL*, as an anti-sense transcript of the *p15* gene in the *INK4* locus [51]. Both our study and other research have revealed that *ANRIL* is involved in epigenetic repression of the transcription of the *INK4* locus [31, 32] (Table 1). We found that depletion of *ANRIL* by short hairpin RNA (shRNA) decreased the recruitment of SUZ12 to the *INK4* locus and promoted the expression of *p15* gene dramatically and *p16* gene moderately, but had no effect on *ARF* [31]. SUZ12 is a component of the PRC2 complex. In contrast, Yap et al. [32] demonstrated that *ANRIL* binds to CBX7, a component of the PRC1 complex, in the chromatin fraction, and recruits PRC1 to the *INK4* locus to mediate transcriptional suppression. Therefore, *ANRIL* binds to the PRC2 complex to recruit it to the *INK4* locus, and then histone H3K27 methylation is mediated by EZH2 in the PRC2 complex. Next, PRC2 with *ANRIL* is recognized by CBX7, and the PRC1 complex is recruited to the region. Further, histone H2AK119 monoubiquitination is induced to repress transcription of the *INK4* locus. Moreover, we demonstrated that depletion of *ANRIL* promotes growth arrest and induces senescence-associated beta-galactosidase in WI38 human fibroblasts [31]. Yap et al. [32] also suggested that CBX7-mediated suppression of the *INK4* locus is involved in regulating cellular senescence. These reports strongly suggest that *ANRIL* participates not only in cell proliferation but also in suppressing premature senescence via the recruitment of PRC1 and PRC2 to the *INK4* locus (Fig. 3a).

It is important to understand how *ANRIL* expression is regulated. We found that excess RAS signaling promoted by the introduction of activated H-RasG12V into WI38 fibroblasts suppressed *ANRIL* expression and induced p15 and p16, thereby arresting the cell cycle and inducing senescence-associated beta-galactosidase [31] (Fig. 3b). Recently, Wan et al. [52] reported that *ANRIL* is induced by DNA-damaging agents via the ATM-E2F1 pathway, but p53 is not induced. Moreover, they suggested that depletion of *ANRIL* decreases homologous recombination after DNA double-strand breaks, although it is unclear whether *ANRIL* promotes DNA repair via the recruitment of PRC1 and PRC2 to the *INK4* locus. Further studies are required on *ANRIL* function in response to cellular stresses. Moreover, Yang et al. found that *lncRNA*-*HEIH* is highly expressed in HBV-related hepatocellular carcinoma. It negatively regulates the expression of CDK inhibitors, such as p15, p16, p21, and p57, via interacting with EZH2, and then plays an important role in G0/G1 arrest [53] (Table 1).

p18^ink4c^ (hereafter p18) is another INK4 family CDK inhibitor that also inhibits both CDK4 and 6 [3, 54]. Recently, *ink4c*−/− mice have been shown to develop spontaneous pituitary adenomas [55], the frequency of which is enhanced by deletion of other CDK inhibitor genes [56]. The combined deletion of the *p18* gene (*CDKN2C*) with the *p16* gene is also found in human cancers [57]. Moreover, the expression levels of p16 and p18 are often inversely correlated during the progression of senescence [58]. It has been reported that transcription of the *p18* gene is regulated by Menin-RET-signaling and the PI3K-AKT pathway [59]. Du et al. [60] reported that the lncRNA, *HULC*, negatively regulates the expression of *p18* gene, which is located near the region containing *HULC* (Table 1). *HULC* was identified as an lncRNA upregulated in human hepatocellular carcinoma (HCC) [61] that is transcribed in a CREB-dependent manner [62]. Moreover, the expression of p18 is induced and suppressed by depletion and overexpression of *HULC*, respectively. The expression of p18 is inversely correlated with the expression of *HULC* in human HCC tissue specimens. Furthermore, the hepatitis B virus oncogene product, HBx, activates the *HULC* promoter via CREB to suppress the transcription of the *p18* gene by upregulated HULC [60]. Downregulation of the *p18* gene by HBx via *HULC* induction may contribute to the development of HCC, although it is unknown how *HULC* suppresses the transcription of the *p18* gene.

### Cip/Kip family inhibitors

The transcription of *p57* ^Kip2^ gene (*CDKN1C*), which is located at the *KCNQ1* domain, is epigenetically suppressed as an imprinted gene on the paternal chromosome [63]. *KCNQ1OT1* is paternally expressed as an antisense RNA of the *KCNQ1* domain containing *KCNQ1* and the *p57* ^Kip2^ gene [64]. *KCNQ1OT1* functions as a recruiter that associates with the chromatin modifiers, PRC2 and G9a, and recruits them to the *KCNQ1* domain to suppress the transcription of *p57* ^Kip2^ gene (Table 1). As described above, *lncRNA-HEIH* downregulates the expression of not only the INK4 family inhibitors, p15 and p16, but also the Cip/Kip family inhibitors, p21 and p57 [53].

## LncRNAs regulating the pRB pathway

**Fig. 4:**
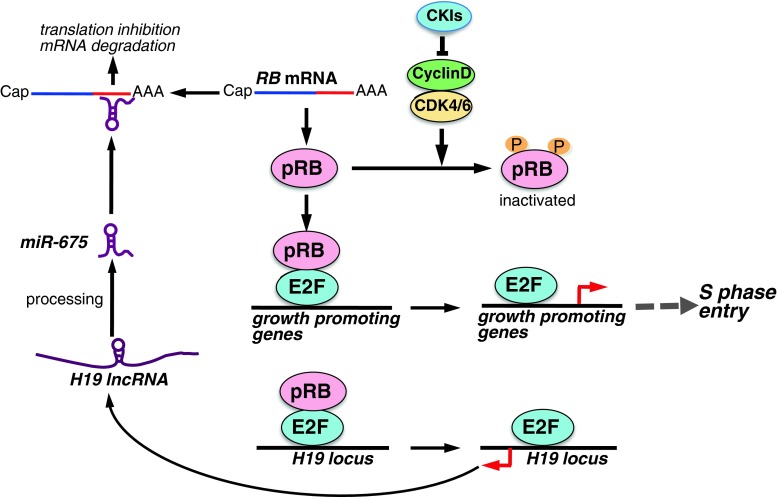
Model showing the proposed mechanisms of lncRNA-mediated regulation of the RB pathway. pRB binds target transcription factors such as E2F and inhibits their activity in the G1 phase. Cyclin Ds-CDK4/6 phosphorylate pRB and activate E2F-mediated transcription in late G1, which regulates the expression of several growth-promoting genes and S phase entry. The transcription of *H19* lncRNA from the *H19* locus is mediated by E2F1. *H19* lncRNA is processed to generate miR-675, which binds to *RB* mRNA and inhibits its translation

As described above, the tumor suppressor pRB is a critical regulator of G1/S progression [7, 65]. It is well known that the expression of the *RB* gene is epigenetically silenced by methylation of the promoter in some cancers, including retinoblastoma [66]. Hypermethylation of the CTCF binding site in the *RB* promoter is mediated by the CTCF protein [67]. CTCF also regulates the expression balance between the *IGF2*/*H19* locus together with DNA methylation of their promoters as an insulator of gene expression [68]. Interestingly, the *H19* gene encodes a 2.9-kb lncRNA, and the *H19* lncRNA is a precursor of miR-675 [69]. The expression of *H19* lncRNA is mediated by E2F1 and promotes cell proliferation [70], but the mechanism is unknown. Tsang et al. [71] reported that the *H19* lncRNA-derived miR-675 associates with the 3′ untranslated region of *RB* mRNA to negatively regulate pRB expression (Fig. 4; Table 1). In human colorectal cancer, *H19* lncRNA/miR-675 expression is inversely correlated with pRB expression [71]. Therefore, *H19* lncRNA/miR-675 may be a critical negative regulator of the RB tumor suppressor pathway (Fig. 3). Moreover, pRB suppresses E2F-dependent transcription of *H19* transcription via repression of the *H19* promoter. Therefore, the *H19*-*RB* axis is self-regulated.

### LncRNAs regulating the p53 pathway

**Fig. 5:**
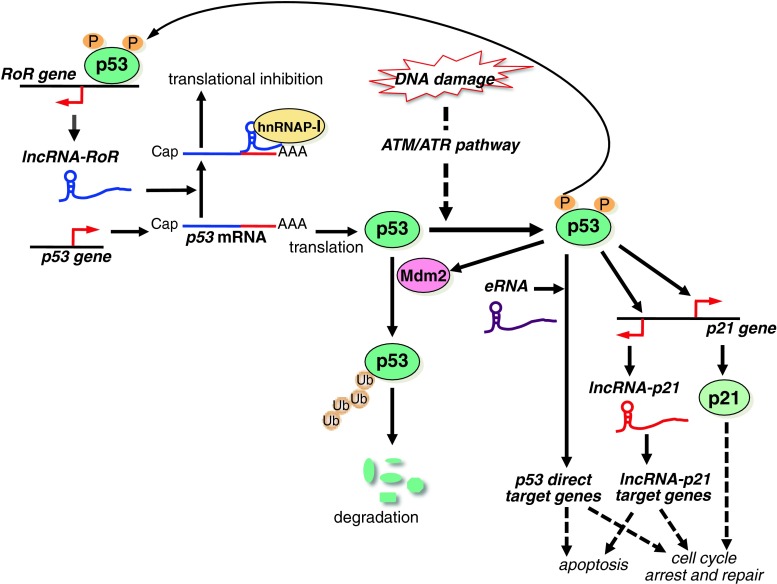
Model showing the proposed mechanisms of lncRNA-mediated regulation of the p53 pathway. p53 controls cell cycle arrest, repair, and apoptosis in response to DNA damage. *lncRNA*-*RoR* binds to hnRNP-I and collaboratively suppresses p53 mRNA translation. This is an autoregulatory feedback loop that controls p53 levels. In response to DNA damage, p53 is stabilized and activated via phosphorylation mediated by the ATM/ATR pathway. p53 directly binds the target genes and regulates their expression to control cell cycle arrest, repair, and apoptosis. *eRNAs* are involved in promotion of p53-target genes in p53-dependent cell cycle arrest. p21 and *lncRNA*-*p21*, which is transcribed near the *p21* ^*Cip1*^ gene, are p53-target genes. *lncRNA*-*p21* controls the expression of some p53-target genes. p53 function is partially mediated by gene regulation via *lncRNA*-*p21*

Another important tumor suppressor, p53, functions as the gatekeeper of the genome to control cell cycle arrest and apoptosis in response to DNA damage [65, 72]. Although p53 is unstable, it is stabilized and activated via phosphorylation mediated by the ATM/ATR pathway in response to DNA damage. Moreover, p53 is also regulated via phosphorylation at various sites by specific kinases [73]. Zang et al. [74] reported that *lncRNA*-*RoR* negatively regulates p53 expression, thereby suppressing doxorubicin-induced G2/M arrest and apoptosis (Table 1). Depletion of *lncRNA*-*RoR* leads to p53 accumulation, and overexpression of *lncRNA*-*RoR* suppresses p53 expression. *LncRNA*-*RoR* binds to phosphorylated heterogeneous nuclear ribonucleoprotein I (p-hnRNP-I) in cytoplasm and thereby suppresses p53 translation. The 28-base RoR sequence is sufficient for its function. Additionally, wild-type p53 binds to the *RoR* promoter to promote transcription of *lncRNA*-*RoR*, but mutant p53 does not bind to this promoter. This is a novel autoregulatory feedback loop that controls p53 levels (Fig. 5).

Recently, Melo et al. [75] reported that enhancer RNAs (*eRNAs*) are required for coordinated promotion between p53 target genes and p53-bound enhancer regions distant from the target gene, and participate in p53-dependent cell cycle arrest (Table 1). LncRNA *loc285194* was suggested to have a tumor suppressor function, but its mechanism was unknown. Liu et al. found that *loc285194* is induced by binding of p53 to its binding site in the promoter (Table 1). Moreover, they indicated that *loc285194* binds to and inhibits miR-211, thereby downregulating miR-211-mediated cell proliferation [76]. *Loc285194* is downregulated in human colon cancer specimens, and thus may contribute to the tumor suppressive function of p53 to inhibit miR-211 [76].

Huarte et al. [77] identified *lncRNA*-*p21*, which is transcribed near the *p21* ^Cip1^ gene (*CDKN1A*) as a p53-target gene. p53 directly binds to its binding element in the *lncRNA*-*p21* promoter. Depletion of *lncRNA*-*p21* alters the expression of some p53-target genes except for *p21* gene and inhibits apoptosis (Fig. 5; Table 1). *lncRNA*-*p21* binds to hnRNP-K and recruits it to the target genes, but the mechanism of target gene regulation is unknown. p53 function is partially mediated by gene regulation via *lncRNA*-*p21*-hnRNP-K. Moreover, Yoon et al. proposed that *lncRNA*-*p21* functions as a modulator of translation. *lncRNA*-*p21* associates with target mRNAs such as β-catenin and JunB in collaboration with Rck/p54 RNA helicase, and thus the translation of the target mRNAs is repressed [78]. Therefore, *lncRNA*-*p21* regulates both transcription in the nucleus and translation in the cytoplasm.

*PANDA* (p21-associated ncRNA DNA damage-activated) was identified as a *p21* promoter-derived transcript using ultra-high density tiling array of 56 cell-cycle genes. It is induced by DNA damage in a p53-dependent manner [79] (Table 1). *PANDA* binds to and inhibits NF-YA transcription factor, which limits the expression of proapoptotic genes such as *FAS* and *BIK* and results in the repression of apoptosis. *PANDA* is selectively induced in metastatic ductal carcinomas but not in normal breast tissue [79]. The results suggest that abnormal overexpression of *PANDA* may suppress apoptosis induced by DNA damage, which will accumulate and push the genome toward carcinogenesis.

## Perspectives

Although the mechanisms of cell cycle regulation via cyclin–CDK, the p53/RB pathway, and the checkpoint pathway have been described in detail, recent studies on lncRNAs strongly suggest that lncRNAs control the expression of cell cycle regulators. Therefore, lncRNAs are critically involved in cell cycle regulation. However, it is unclear why lncRNAs might be deployed to regulate the cell cycle. As described in the “Introduction”, lncRNAs involved in cell cycle regulation are classified into four groups. As shown in Table 1, *ANRIL*, *lncRNA*-*HEIH*, and *KCNQ1OT1* are involved in epigenetic regulation of target gene transcription by collaborating with chromatin modifiers, which are classified as epigenetic regulators. *ncRNA*_*CCND1*_, *SRA, PANDA*, and* lncRNA-p21* directly interact with the transcriptional machinery on the target genes and collaboratively regulate transcription as transcription factor regulators. Post-transcription regulators including *gadd7, MALAT1, lncRNA*-*RoR*, and *loc285194* bind to their specific target mRNA to suppress translation and/or to modulate mRNA stability. *SRA* and *MALAT1* also promote protein–protein interactions and are classified as protein scaffolds. Because the general cell cycle is closely associated with various cellular events as well as biological processes, it should be accurately regulated. Post-transcription regulators such as *gadd7*, *MALAT1*, *H19 lncRNA*, and *loc285194* can rapidly and transiently suppress translation of their target genes. Transcription factor regulators such as *ncRNA*_*CCND1*_, *SRA*, *PANDA*, and *lncRNA*-*p21* that directly interact with the transcription machinery on the target genes may also participate in transient regulation. Alternatively, epigenetic regulators such as *ANRIL*, *lncRNA*-*HEIH*, and *KCNQ1OT1* may have long-term effects on cellular senescence and imprinting because they mediate epigenetic regulation of cell cycle regulatory genes via chromatin modifiers. From this viewpoint, the cell cycle-regulated lncRNAs mainly control cellular levels of cell cycle regulators via various mechanisms, and may provide diversity and reliability to the general cell cycle.

It is interesting that many lncRNAs are associated with the DNA damage response. As shown in Table 1, 4 of 14 lncRNAs, *lncRNA*_*CCND1*_ *, gadd7, ANRIL* and *PANDA*, are induced by DNA damage. Another 4 lncRNAs, *lncRNA*-*RoR, lncRNAp21, p53*-*induced eRNA*, and *loc285194*, are induced in a p53-dependent manner, suggesting that they are induced by DNA damage. Therefore, these reported lncRNAs may participate in cell cycle arrest or induction of apoptosis as non-canonical DNA damage responses, whereas the ATM/ATR pathway is involved in a canonical DNA damage response to inactivate CDK activity as a DNA damage checkpoint. LncRNAs-mediated non-canonical pathways may ensure the response to DNA damage is diverse and reliable depending on the cellular context.

Considering the recent progress in lncRNA research, many lncRNAs that have a functional role in cell cycle regulation remain to be identified because the functions of only a small percentage of the total lncRNA population are understood. To clarify the roles of lncRNAs in cell cycle regulation, it should be determined how they regulate the target cell cycle regulators and which signaling pathways induce these lncRNAs. Since abrogation of the cell cycle is closely associated with cancer development and growth, cell cycle regulatory lncRNAs such as *ANRIL* and *PANDA* may have oncogenic properties. The importance of lncRNAs in cell cycle regulation will be clarified by further pathological studies. Moreover, these cell cycle regulatory lncRNAs may be novel candidate molecular targets for cancer therapy or diagnosis.
